# Illumina^®^ Sequencing Reveals Candidate Genes of Carotenoid Metabolism in Three Pummelo Cultivars (*Citrus Maxima*) with Different Pulp Color

**DOI:** 10.3390/ijms20092246

**Published:** 2019-05-07

**Authors:** Cui-Cui Jiang, Yan-Fang Zhang, Yan-Jin Lin, Yuan Chen, Xin-Kun Lu

**Affiliations:** 1 Fruit Research Institute, Fujian Academy of Agricultural Sciences, Fuzhou 350013, China; cuiliao2046@163.com (C.-C.J.); zhangyanfangyes@sina.com (Y.-F.Z.); lyj0591@163.com (Y.-J.L.); 2 Institute of Agricultural Engineering and Technology, Fujian Academy of Agricultural Sciences, Fuzhou 350003, China; katecy@163.com

**Keywords:** *Citrus maxima*, transcriptome, carotenoid biosynthesis, transcription factor

## Abstract

Pummelo (*Citrus maxima*) is one of important fruit trees, which belongs to *Citrus* species. The fruits of different pummelo cultivars have different colors and differ in the contents of carotenoid. Our results clearly showed that ‘Huangjinmiyou’ (HJMY) has the highest content of β-carotene, followed by ‘Hongroumiyou’ (HRMY) and ‘Guanximiyou’ (GXMY). Lycopene is dominantly accumulated in HRMY. However, the molecular mechanism underlying the carotenoid accumulation in pummelo flesh is not fully understood. In this study, we used the RNA-Seq technique to investigate the candidate genes of carotenoid metabolism in the flesh of pummelo cv. GXMY and its mutants HRMY and HJMY in three development periods of fruit. After data assembly and bioinformatic analysis, a total of 357 genes involved in biosynthesis of secondary metabolites were isolated, of which 12 differentially expressed genes (DEGs) are involved in carotenoid biosynthesis. Among these 12 DEGs, phytoene synthase (*PSY2*), lycopene β-cyclase (*LYCB2*), lycopene Ɛ-cyclase (*LYCE*), carotenoid cleavage dioxygenases (*CCD4*), 9-cis-epoxycarotenoid dioxygenase (*NCED2*), aldehyde oxidase 3 (*AAO3*), and ABA 8′-hydroxylases (*CYP707A1*) are the most distinct DEGs in three pummelo cultivars. The co-expression analysis revealed that the expression patterns of several transcription factors such as *bHLH*, *MYB*, *ERF*, *NAC* and *WRKY* are highly correlated with DEGs, which are involved in carotenoid biosynthesis. In addition, the expression patterns of 22 DEGs were validated by real-time quantitative PCR (RT-qPCR) and the results are highly concordant with the RNA-Seq results. Our results provide a global vision of transcriptomic profile among three pummelo cultivars with different pulp colors. These results would be beneficial to further study the molecular mechanism of carotenoid accumulation in pummelo flesh and help the breeding of citrus with high carotenoid content.

## 1. Introduction

Carotenoid is the second most abundant compound in natural pigments and provides colorless, yellow, orange, and red colors for many fruits, flowers and vegetables [1]. Plant is the main source of carotenoid for human. In recent years, with the improvement of people’s living standards and health consciousness, horticultural products for high carotenoid content have been favored by many consumers. Therefore, it is critical and urgent to accelerate horticultural plant breeding for high carotenoid content through breeding technologies.

*Citrus* takes the leading position in fruit industry worldwide. Color of citrus flesh represents an essential fruit quality attribute. Recently, high demand for citrus with different flesh colors provides potentials for genetic improvement of citrus. It is clear that coloration of most citrus fruits is mainly caused by the accumulation of carotenoid [2,3,4]. Composition and content of carotenoid in fruits depend on cultivars and species [5]. In order to regulate coloration of citrus fruit genetically, several novel methods, such as gene cloning and transcriptomic and proteomic analysis, are used to isolate genes associated with carotenoid metabolism [2,6]. Until now, RNA-Seq has been successfully applied in fruit development of *Citrus* [7,8,9,10]. Previous studies mostly focused on the content and composition of carotenoid in sweet orange (*C. sinensis* L. Osbeck), satsuma mandarin (*C. unshiu* Marc.), and lemon (*C. limon* Burm.) [2]. However, the carotenoid regulation mechanism in pummelo flesh was not well understood. Pummelo is monoembryonic, and for this reason, its true hybrids are easily obtained by cross-breeding without nucellar seedling in progenies. It is helpful to identify genetic control of carotenoid metabolism with pummelo as materials. Moreover, pummelo, one of three basic species of citrus, shows lower heterozygosity than sweet orange, so it can overcome heterozygous loci in genetic analysis. Liu et al. [11] investigated the carotenoid profile of red-flesh pummelo cv. “Chuhong” and pale green-flesh pummelo cv. “Feicui” by high-performance liquid chromatography (HPLC) and GC/MS and concluded that there are different tissue-biased patterns of carotenoid biosynthesis in pummelos with different flesh colors. Guo et al. [10] identified the differentially expressed genes in GXMY and its orange-pericarp mutant using the RNA-Seq technique and revealed extensive changes of gene expression level between the control and its mutant. There was no report on the molecular mechanism of carotenoid accumulation in pummelo flesh.

Since 1998, we have discovered two mutants with different flesh colors from GXMY in Pinghe County, Zhangzhou City, China. These mutants can be used as materials to study the molecular mechanism of carotenoid accumulation. *C. maxima* cv. GXMY is traditionally famous fruit in China and has yellow flesh, in which the total carotenoid content is 0.875 μg/g in fresh weight (FW) [12]. HRMY is a bud mutant of GXMY with red flesh, of which the total carotenoid content is 96.55 μg/g FW [12]. HJMY is also a high-quality bud mutant of GXMY with golden yellow flesh, of which the total carotenoid content is 251.77 μg/g FW [12,13]. In order to unveil the molecular mechanism underlying different colorations of GXMY and its mutants HJMY and HRMY, we investigated the gene expression and transcription factors associated with carotenoid metabolism in the flesh of pummelo cv. GXMY, HJMY and HRMY in three key stages of fruit development (according to the pigment contents and the ratio of pulp and fruit) by using the RNA-Seq technique. The identified genes could be useful for studying the molecular mechanism of carotenoid accumulation in citrus flesh and help the breeding of citrus with high carotenoid content.

## 2. Results

### 2.1. Changes in β-Carotene and Lycopene Contents During Fruit Development of Three Different Pummelo Cultivars

**Figure 1:**
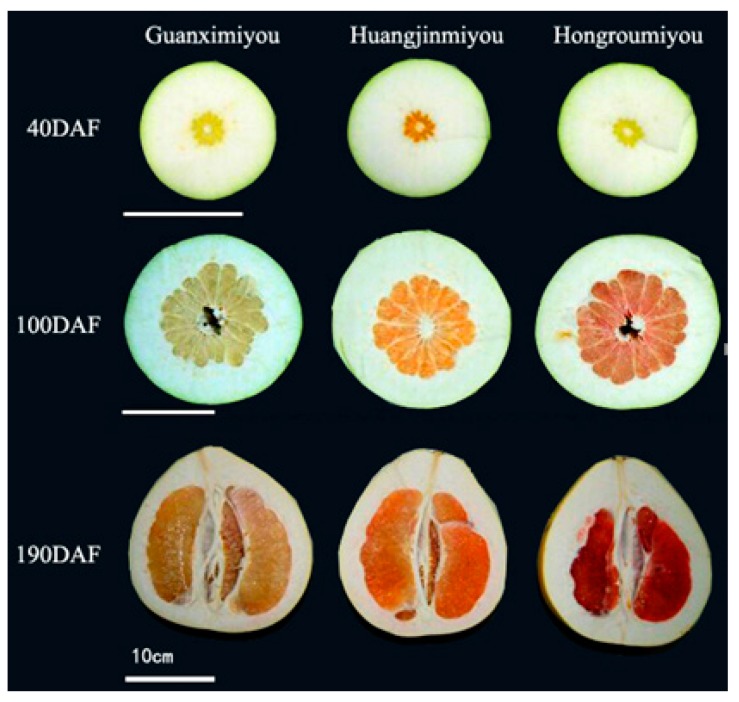
Three pummelo cultivars at different periods of fruit growth and development.

**Figure 2:**
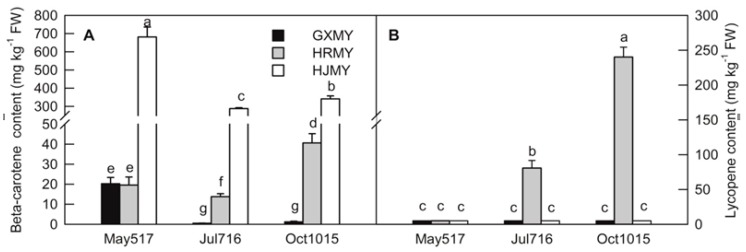
β-carotene and lycopene contents in three pummelo cultivars of different fruit growth and development periods. Bars represent means ± SE (*n* = 3). Differences among the samples were analyzed by three (cultivars) × three (sampling dates) ANOVA. Different letters indicate a significant difference at *p* < 0.05.

The appearances of three pummelo cultivars of different fruit growth and development periods are presented in Figure 1. The contents of β-carotene and lycopene in three different pummelo cultivars were measured by HPLC (Figure 2). As results indicated, HJMY has the highest content of β-carotene with a value of about 681.45 mg/kg FW, almost 34 times higher than that in GXMY and HRMY at an early development stage 40 days after flowering (DAF). The β-carotene content decreases at 100 DAF and then increases at 190 DAF in HJMY and HRMY, whereas it decreases during the entire development of GXMY. Lycopene was detected only in the HRMY, showing an increasing trend with the development of the fruit and mainly accumulated in the late stage of fruit development. Based on the overall trends, HJMY has the highest content of β-carotene, followed by HRMY and GXMY, and lycopene is dominantly accumulated in HRMY.

### 2.2. Transcriptome Sequence and De Novo Assembly

**Figure 3:**
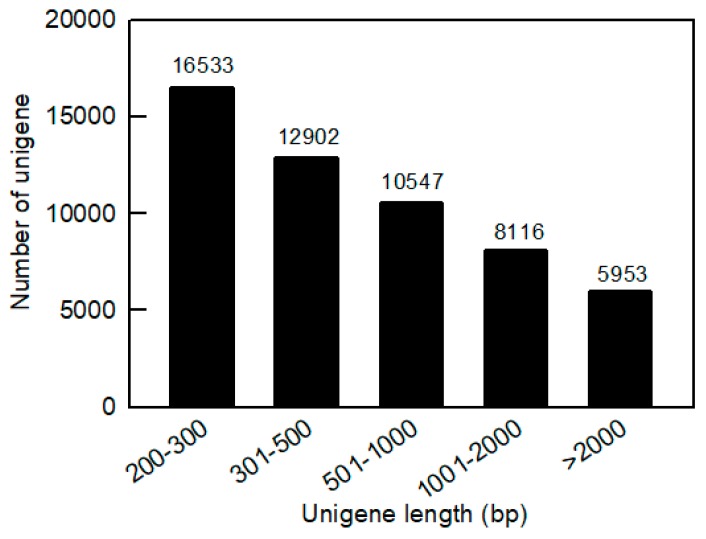
Sequence length distribution of the unigenes in fruit transcriptomes of three pummelo cultivars. The x-axis indicates the unigene length interval from 200 bp to >2000 bp. The y-axis indicates the number of unigenes of each given sequence length.

Nine cDNA libraries were constructed from the total RNA of three pummelo cultivars at 40 DAF, 100 DAF, and 190 DAF. The original sequencing data were filtered and assembled as shown in Table 1. The results showed that 201.78 million high-quality reads were obtained with a total base number of 40.66 billion (giga or G) and a total of 17.65 million contigs were assembled. The Q30 value (base call accuracy of each read is more than 99.9%) of clean reads in each library is more than 85%. These libraries were subjected to RNA-Seq using an Illumina HiSeq2500 and the clean reads from different libraries are presented in Table 1. Using Trinity software, the clean reads from the nine libraries were assembled into 106,957 transcripts with an average length of 858 bp and 54,051 unigenes with a mean length of 858 bp (Table 1). The size distributions of these unigenes are shown in Figure 3. Among the unigenes, the lengths of 12,902 unigenes (23.87% of the total number of unigenes of 54,051) range from 300 to 500 bp, the lengths of 10,547 unigenes (19.51% of the total number of unigenes) range from 501 to 1000 bp, the lengths of 8116 unigenes (15.02% of the total number of unigenes) range from 1000 to 2000 bp, and the lengths of 5953 unigenes (11.01% of the total number of unigenes) are more than 2000 bp.

### 2.3. Functional Annotation and Classification of Unigenes

**Figure 4:**
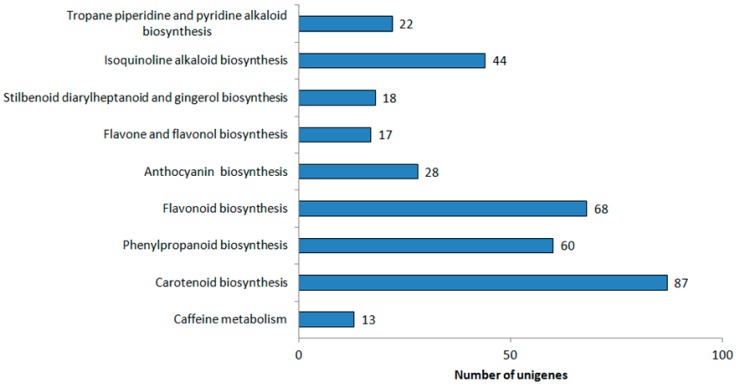
Classification based on “Biosynthesis of secondary metabolites” categories.

To determine and annotate the transcriptome data of three pummelo cultivars, approximately 24,206 unigenes were searched against five public databases [NCBI non-redundant protein (Nr), Swiss-Prot, Kyoto Encyclopedia of Genes and Genomes (KEGG), Clusters of Orthologous Groups (COG), and Gene Ontology (GO)] with an E-value of ≤ 10^−5^ by BLASTX and BLASTN searching engines [14]. The functional annotation results showed that there were 8876, 17,764, 6093, 16,857, and 24,125 unigenes annotated to COG, GO, KEGG, Swiss-Prot, and Nr, respectively. Totally, only 44.8% of the unigenes (24,206) could be successfully annotated to these five public databases. The remaining unigenes (55.2% of the total number of unigenes) have no homologs with the known genes, which is likely due to the presence of short sequences. Pathway-based analysis can help to understand the biological functions and interactions of genes. There were 6093 unigenes assigned to 117 KEGG pathways (24,206) (Table S1, Supplementary Materials). The majority of unigenes were associated with metabolic pathways (1847). We focused on the “Biosynthesis of secondary metabolites” category in relation to fruit pigmentation. In this category, 357 unigenes were classified into nine subcategories (Figure 4). Among these nine subcategories, “Carotenoid biosynthesis” is the highest clustered, followed by “Flavonoid biosynthesis”. “Flavone and flavonol biosynthesis” and “Caffeine metabolism” appear to be the smallest groups.

### 2.4. Analysis of DEG During Fruit Development of Three Different Pummelo Cultivars

**Figure 5:**
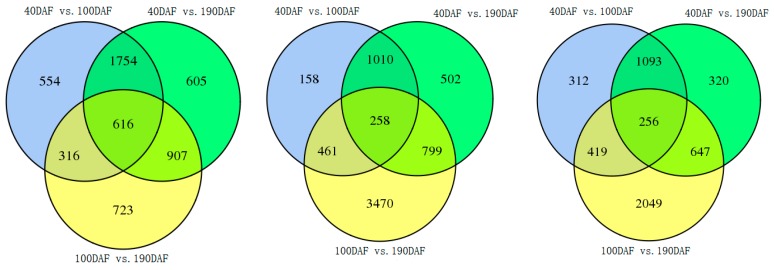
Venn diagram illustrating the number of unigenes expressed of three pummelo cultivars at different developmental stages.

Using the criteria of *p* < 0.01 and |log_2_ (fold change)| ≥ 2, a differential expression analysis of unigenes was performed during fruit development of three different pummelo cultivars. The relationships between different DEG groups are displayed as Venn diagram (Figure 5), and 616, 258, and 256 unigenes were shared in all development stages of three pummelo cultivars, respectively. Through comparing the libraries, we found a number of differentially related genes, as shown in Table 2. The results suggested that the developmental periods with the most dynamic changes in the transcriptome are between 40 DAF and 190 DAF in GXMY, and between 100 DAF and 190 DAF in HRMY and HJMY.

### 2.5. Candidate Genes Involved in Carotenoid Biosynthesis

**Figure 6:**
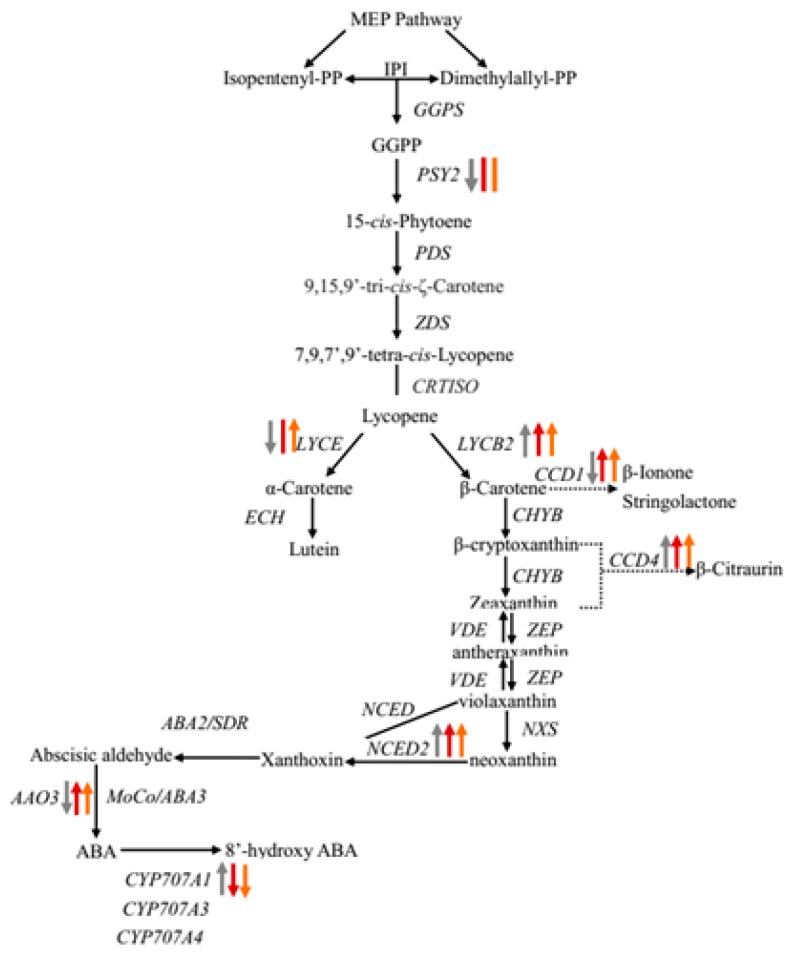
Diagram showing a proposed model for carotenoid biosynthesis of three pummelo cultivars. Biosynthesis pathways are shown with solid arrows and regulatory interactions are shown with broken arrows. Up arrow (↑), down arrow (↓), and vertical bar (|) indicate significantly up-regulated, down-regulated and no obvious change of gene expression of these enzymes, respectively. The colored arrows or bars represent the change of gene expression in GXMY (gray), HRMY (red), and HJMY (golden).

To further search for the genes involved in carotenoid biosynthesis, we analyzed the transcriptomic data of three pummelo cultivars at different developmental stages. Based on the KEGG annotation, 12 DEGs that encode enzymes related to carotenoid biosynthesis pathway were isolated (Table 3 and Table S2 (Supplementary Materials); Figure 6).

Transcription factors (TFs) are key regulators of gene expression associated with various aspects of fruit growth and development including carotenoid biosynthesis. In order to identify differentially expressed transcription factors from three pummelo cultivars, a BLASTX search was conducted using all the transcription factors sequences available in the PlnTFDB database. In total, 435 unigenes (Table S3, Supplementary Materials) were predicted to encode transcription factors from 53 different families (Table S4, Supplementary Materials) and 227 of them were differentially expressed (Table S5, Supplementary Materials). A transcription abundance correlation analysis was carried out between the differentially expressed transcription factors and structural genes from the carotenoid biosynthesis pathway. The expression levels of the 48 transcription factors are highly correlated with those of the candidate structural genes (Table S6, Supplementary Materials). The identified transcription factors included the transcription factors that are implicated in regulating carotenoid biosynthesis, such as bHLH, ERF, and MYB (Table 4). The 34 bHLH were differentially expressed during fruit development stages of three pummelo cultivars and nine of them [c11225.graph_c0 (homologous to CsbHLH107), c12645.graph_c0 (homologous to BIM1 isoform X1), c21405.graph_c0 (homologous to CsbHLH162), c22086.graph_c0 (homologous to CsbHLH57), c2272.graph_c0 (homologous to CsbHLH35), c22910.graph_c0 (homologous to CsbHLH61), c27501.graph_c0 (homologous to CsbHLH130), c6149.graph_c0 (homologous to ILR3), and c589.graph_c1 (homologous to HEC2)] were associated with the carotenoid biosynthesis pathway (Table 4, Tables S3 and S6 (Supplementary Materials)). Eight of the differentially expressed transcription factors annotated as ERF or AP2/ERF showed a significant positive correlation with carotenoid biosynthesis-related genes (Table 4 and Table S6 (Supplementary Materials)). Although they are correlated with none of the structural genes, the expression levels of the homolog of *CsARF19* (c23948.graph_c1) and *CsARF5* (c26184.graph_c0) show a differential expression during fruit development of three different cultivars. Four of the differentially expressed transcription factors annotated as MYB show a significant positive correlation with carotenoid biosynthesis-related genes (Table 4 and Table S6 (Supplementary Materials)), while only two of the *MYB* genes (c22501.graph_c0 and c23847.graph_c0) show a negative correlation with the expression of structural genes. A *MYB* gene (c21421.graph_c0), which shares a high similarity with transcription factor AS1 (XP_006429090.1) from *C. clementina*, significantly correlates with six carotenoid biosynthesis-related structural genes (Table S6, Supplementary Materials). In addition, one *NAC* (c13516.graph_c0) and two *WRKY*s (c21980.graph_c0 and c34311.graph_c0), which share a high sequence similarity with the NAC domain-containing protein 72 (XM_006451877.2), *WRKY22* (XM_0064444887.2), and *WRKY17* (XM_006450132.2) from *C. clementina*, respectively, were also found to be correlated to the genes involved in the carotenoid pathway.

### 2.6. Real-Time Quantitative PCR (RT-qPCR) Analysis of Selected DEGs During Fruit Development of Three Pummelo Cultivars

**Figure 7:**
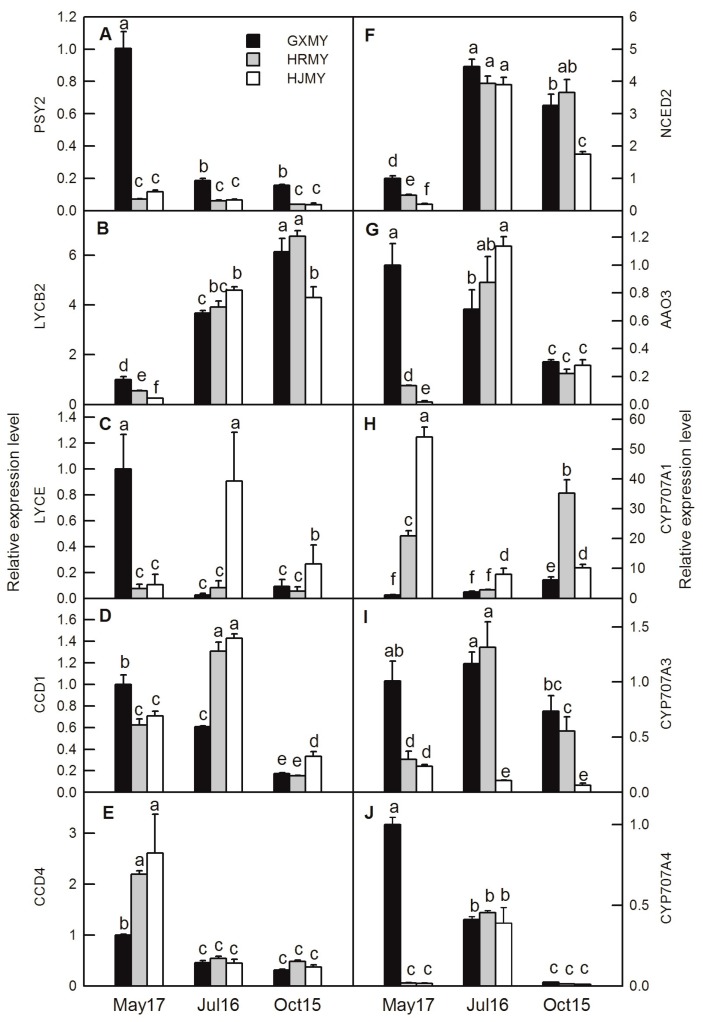
Gene expression level analysis of 10 differentially expressed structural genes related to carotenoid biosynthesis of three pummelo cultivars at different developmental stages. Bars represent means ± SE (*n* = 3). Differences among the samples were analyzed by three (cultivars) × three (sampling dates) ANOVA. Different letters indicate a significant difference at *p* < 0.05.

**Figure 8:**
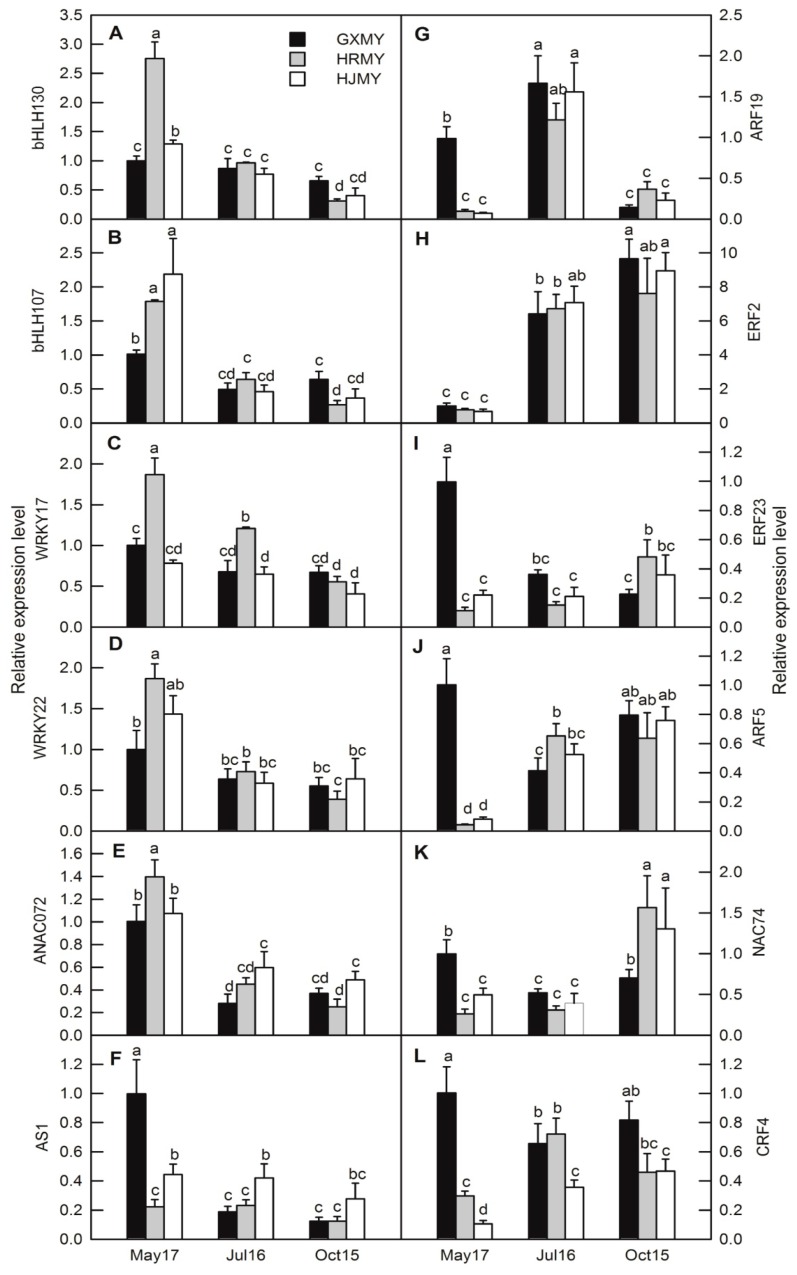
Expression analysis of 12 differentially expressed transcription factors related to carotenoid biosynthesis of three pummelo cultivars at different developmental stages. Bars represent means ± SE (*n* = 3). Differences among the samples were analyzed by three (cultivars) × three (sampling dates) ANOVA. Different letters indicate a significant difference at *p* < 0.05.

To further confirm the results of transcriptomic analysis, 11 enzyme-encoding genes and twelve transcription factors associated with carotenoid biosynthesis were analyzed by RT-qPCR in three pummelo cultivars. As RT-qPCR indicated, all selected DEGs were expressed at varying levels during the three stages and their RNA-Seq data have a close correlation (Figure 7; Figure 8).

## 3. Discussion

Carotenoids are the main pigments in citrus fruits and different carotenoid components determined different colors in citrus fruits. Carotenoid has an important role in the sensory quality and nutritional quality of citrus fruit. Our previous studies have shown that the colorful pigments of HJMY juice are mainly due to the contents of β-carotene, phytoene, α-carotene, and β-cryptoxanthin, and the total amount of carotenoid reaches 251.77 μg·g^−1^·FW, which are 2.6 and 287.7 times those of HRMY and GXMY, respectively [12]. This study showed that the β-carotene and lycopene are dominantly accumulated in HJMY and HRMY during the development of pummelo fruits, respectively, which confer the golden orange color in HJMY and red color in HRMY (Figure 1 and Figure 2).

Identification and profile of the members of gene families involved in the carotenoid pathway could explain the diversity of coloration in fruit. Previous studies have revealed that the key regulatory genes, such as phytoene synthase (*PSY*), lycopene β-cyclase (*LCYB*), lycopene ε-cyclase (*LCYE*), *CCD* and *NCED* in carotenoid biosynthesis, might be key factors in controlling the carotenoid metabolism (Figure 6) [15,16,17,18,19,20,21]. The PSY is the rate-limiting enzyme in carotenoid biosynthesis and plays an important role in the biosynthesis of carotenoid in many plants, such as apple, carrot, and tomato [22,23,24]. *PSY* appears to be a small gene family in most plants including citrus. The up-regulation of *PSY1* gene and accumulation of carotenoid are found in the flavedo of sweet orange [25]. However, there are few reports regarding *PSY2*. Recently, a new *PSY* member (*PSY2*) was reported in sweet orange and its expression is at a lower level than that of *PSY1* in both flavedo and juice sacs in Valencia and Rohde Red Valencia [26], which suggests that *PSY2* may also contribute to the accumulation of carotenoid in citrus fruit. In the current study, the *PSY2* (c27977.graph_c0, Table 3; Figure 7A) has a high expression level at an earlier stage of GXMY and then decreases during the entire fruit development. It has relatively low expression levels in both HRMY and HJMY. According to our pigment measurements, *PSY2* might not be the key regulatory gene responsible for carotenoid biosynthesis and accumulation in pummelo pulp (Figure 2 and Figure 7A). However, the *PSY2* has the highest expression level in yellow apple germplasm, which is significantly positively correlated with carotenoid contents in apple [22].

Previous studies showed that the transcriptional regulation of LYCB and LYCE are the critical regulatory points in carotenoid biosynthesis [27]. In citrus, two LCYB members have also been isolated in navel orange fruit and grapefruit cultivars, “marsh” (white flesh) and “flame” (red flesh) [28]. Two *LCYE* genes are identified in sweet orange tissues, but only one *LCYE* gene is detected in leaves and fruits [29]. The expression levels of *VvLCYB* and *VvLCYE* gradually increase with the accumulation of carotenoid contents during the ripening of grapevine fruit [18]. In sweet potatoes, over-expression of *IbLCYB2* significantly increases the carotenoid contents and enhances abiotic tolerance by positively regulating the biosynthesis of carotenoid and ABA [30]. The expression level of *LCYB2* increases markedly during the ripening of papaya [31]. Here, our results showed that the expression level of *LYCB2* increases from 40 DAF to ripening stages (190 DAF) in all the three pummelo cultivars with a slightly lower expression level in HJMY at the ripening stage (190 DAF) (Table 3; Figure 7B). The down-regulation of LYCE is only found at the development of GXMY, whereas the expression level of *LYCE* is firstly up-regulated and then up-regulated during fruit development in HRMY and GXMY (Table 3; Figure 7C). The expression levels of LYCE are higher in HRMY and HJMY at the color break period (100 DAF) and ripening period (190 DAF) than in GXMY, which may contribute to the high α-carotene content in HRMY and HJMY pulp [12].

In plants, the CCD family consists of nine members, which catalyze the oxidative cleavage of carotenoid [32]. Among them, five genes are related to the synthesis of ABA, named *NCED* (*NCED2*, *3*, *5*, *6*, *9*), and the remaining four are named *CCD1*, *4*, *7*, *8*, which are closely related to the growth and development of lateral branches, plant hormones (such as strigolactones), the formation of volatile aromatic compounds, and colorless or red volatile apocarotenoids compounds [33]. Carotenoid accumulation in several yellow fruits and flowers has been shown to be negatively associated with *CCD1* or *CCD4* expression. For example, transcription inactivation of *CmCCD4a* causes yellow coloration of flowers in chrysanthemum [34]. In azalea petals, the expression levels of *CCD4* are higher in ‘Miyamasatsuki’ (white-flowered) and the progeny than in *R. japonicum* f. *flavum* (yellow-flowered) at all stages [35]. In fruit, the negative correlation between the expression level of *CCD4* and the carotenoid content is also observed in summer squash, peach, potato, and apple [36,37,38,39]. Here, we also found that the expression level of *CCD4* (c20564.graph_c0) gene, which is homologous to the *CCD4a* in *C. clementina* (DQ309330.1), decreases with the fruit development in three pomelo varieties (Table 3; Figure 7E). In addition, we also found that the expression level of the *CCD1* (c29184.graph_c0) increases from the early stages of fruit development (40 DAF) to the color break period (100 DAF), followed by a decrease as fruit becomes mature in HJMY and HRMY. In GXMY, the expression level of *CCD1* decreases as the fruit becomes mature (Table 3; Figure 7D).

ABA is synthesized in the plastids from xanthophyll pigments and is derived from a precursor generated by the carotenoid biosynthesis pathway [40]. Previous research has demonstrated that ABA could potentially regulate carotenoid composition during ripening by increased leaf chlorophylls, carotenoid, and fruit LYCO in tomato [41]. Furthermore, foliar-applied ABA on grapes (*Vitis vinifera*) results in stimulatory effects on fruit color [42]. Previous researches indicated that the cleavage reaction, catalyzed by NCED, is the first and regulatory step in ABA biosynthesis [43,44]. The expression levels of *NCED1* and *NCED3* are down-regulated from 40 DAF to 100 DAF at first and then up-regulated until 190 DAF in three pummelo cultivars, whereas that of NCED2 is up-regulated from 40 DAF to 100 DAF and then down-regulated until 190 DAF (Table 3 Figure 7F). The expression levels of *NCED1* and *NCED3* are higher at both of 40 DAF and 100 DAF in HRMY and HJMY than in GXMY (Table 3), suggesting that the higher expression levels of *NCED1* and *NCED3* may facilitate the biosynthesis of ABA in HRMY and HJMY. Applications of exogenous ABA increase concentrations of tomato leaf carotenoid and fruits, which might be due to the increased activity of key enzymes such as BC hydroxylase and PSY3 or both carotenoid, and chlorophylls are derived from the MEP pathway [45]. Sun et al. [46] suggested that carotenoid is the precursor of ABA synthesis, which could trigger ethylene production and then induce an increase in carotenoid biosynthesis in the pre-ripening stage of fruit. Conversely, ABA production can also be achieved by the change of carotenoid content and composition [47]. Evidence showed that the regulation of ABA contents in fruit is mediated by two main early-response catabolic enzyme AAO3 and CYP707A1 [48]. Here, we showed that *AAO3* and *CYP707A3* have a high expression level in the color break period in HJMY and HRMY (Table 3; Figure 7G,I). Expression of *CYP707A1* shows an increasing trend in GXMY and HRMY and a decreasing trend in HJMY, which may involve degrading ABA at the later stages of fruit ripening and potentially preventing the over-accumulation of ABA at this stage. These results indicate the possibility to control carotenoid composition and content during fruit ripening by regulating ABA metabolism. Similarly, ABA is also involved in fruit ripening and affects carotenoid accumulation in tomato and strawberry [49,50]. However, whether phytohormones directly regulate the expression level of key genes in carotenoid biosynthesis pathway remains unknown.

Several transcription factors have been reported to be associated with carotenoid metabolism such as RAP2.2 and PIF1 in *Arabidopsis* [51,52], RIN, TAGL1, ERF6 and HB-1 in watermelon [53], NAC, MYB, AP2/ERF, WRKY, bZIP, and ARF in sweet potato [54]. Furthermore, many genes such as *RIN*, *TAG1*, *ERF6*, *AP2a*, *SGR*, *NOR*, *SlNAC4*, and *PIF* have been shown to encode the respective transcriptional factors that affect the expression levels of genes involved in carotenoid biosynthesis through the regulation of ethylene biosynthesis and signaling [55,56,57]. Lu et al. [4] indicated that CsMADS6 directly regulates *LCYB1* and other carotenogenic genes to coordinately and positively modulate carotenoid metabolism in citrus. bHLH transcription factors have been reported to play an important role in carotenoid biosynthesis regulation under environmental signals [57,58]. In tomatoes, a PRE-like atypical bHLH gene *SlPRE2* negatively regulates carotenoid accumulation during fruit ripening by repressing the expression of *PSY1*, *PDS*, and *ZDS* [59]. In citrus, *CubHLH1* is considered to have a similar function like *Arabidopsis* AIFs, which is functionally characterized as a negative regulator of the brassinolide (BR) signaling pathway and might be directly involved in carotenoid metabolism [58]. Here, our results showed that the expression patterns of two bHLH gene homolog *CsbHLH107* (CsbHLH107, c11225.graph_c0) and *AtFBH4* (CsbHLH130, c27501.graph_c0) negatively correlate with the genes involved in carotenoid biosynthesis (Table S6 (Supplementary Materials); Figure 8A,B). *WRKY* genes are known to participate in growth and developmental processes, the biosynthesis of secondary metabolites as well as biotic and abiotic stress responses through modulation of ABA signaling pathways [60]. Recently, a sweet osmanthus *OfWRKY3* was shown to positively regulate the *OfCCD4* gene through binding to its promoter W-box [61]. Our results indicated that two *WRKY* gene homolog *GhWRKY17* (c34311.graph_c0) and *AtWRKY22* (c21980.graph_c0) positively correlate with the expression level of *CCD4* and negatively correlate with *NCED2* (Table S6 (Supplementary Materials); Figure 8C,D). This result implied that *WRKY* gene could be involved in regulating carotenoid metabolism by modulating ABA signaling pathways, but the specific regulatory mechanisms need to be further studied. NAC proteins have also been reported to be related to carotenoid biosynthesis in tomato and papaya [62,63]. In tomato, over-expression of *SlNAC1* leads to a decline in the contents of carotenoid and ABA, decreasing the synthesis of ethylene [62]. In addition, the SNAC4 (the protein structure of SNAC4 is very similar to that of ANAC072) and SNAC9 positively and negatively regulate fruit ripening and carotenoid accumulation by affecting ethylene and ABA levels [64]. Our results indicated that pummelo homologs of NAC072 (c13516.graph_c0) and NAC74 (c9680.graph_c0) are negatively and positively correlated with genes involved in carotenoid biosynthesis, respectively (Table S6 (Supplementary Materials); Figure 8E,K).

Ethylene plays a critical role in the regulation of carotenoid accumulation and carotenogenic gene expression [65,66]. In some non-climacteric fruits like papayas, CpEIN3a acts as a key transcription factor in the ethylene signal transduction pathway and interacts with CpNAC2 to activate the transcription of a subset of carotenoid biosynthesis-related genes, such as *CpPDS2/4*, *CpZDS*, *CpLCYe*, and *CpCHYb* [67]. As a non-climacteric fruit, ethylene promotes the color of citrus fruits and it increases the transcription of carotenoid biosynthesis-related genes and chlorophyll degradation-related genes [68]. Here, we showed that the *ERF2* (c31821.graph_c0) is increased with the development of these three pummelo fruits, whereas the *ERF23* (c8622.graph_c0) decreases in GXMY and increases in HRMY and HJMY during the development of fruit (Table S5 (Supplementary Materials); Figure 8H,I). This result indicated that *ERF23*-mediated biological regulation of ethylene may have a functional role in carotenoid biosynthesis in HRMY and HJMY. Previous studies showed that MYB transcription factors play important regulatory roles in anthocyanins biosynthesis, which could be responsible for fruit color formation [69,70,71]. Recently, studies have shown that MYB transcription factors might also play a role in carotenoid biosynthesis [72]. In this study, six MYB transcription factors are positively correlated with carotenoid biosynthesis-related genes, while only two pummelo MYB transcription factors (c22501.graph_c0 and c23847.graph_c0) show a negatively correlation with the expression of structural genes involved in carotenoid biosynthesis and one MYB transcription factor (c21421.graph_c0) is positively correlated with six structural genes involved in carotenoid biosynthesis (Tables S5 and S6, Supplementary Materials).

## 4. Material and Methods

### 4.1. Plant Materials

Three pummelo cultivars, named GXMY (yellow pulp) and its bud mutants HRMY (red pulp) and HJMY (golden pulp), were manually harvested from plants grown in the pummelo-producing areas in Pinghe County, Zhangzhou City, Fujian Province, China. Fruit samples were picked at 40 DAF (young fruit; the pulp radius accounts for one-fourth of the fruit radius), 100 DAF (color break period; the pulp radius accounts for half of the fruit length), 190 DAF (ripe stage, harvested for commercial sale) from three trees on a clear day, respectively (Figure 1). All the trees were grown in a square of 4 m × 5 m and received a standard horticultural practice such as irrigation, sod cultivation, bagging, and pathogen prevention. Samples were collected at sunny noon, peeled, and sliced into appropriate pieces after their weight and diameter were measured. In all cases, there were three biological replicates per cultivar for each sampling date and each biological replicate was mixed from at least five fruits. The pulp pieced from each fruit or several fruits were wrapped into one aluminum foil bag and labeled. Then, the samples were frozen in liquid nitrogen and stored at −80 °C until analysis.

### 4.2. β-Carotene and Lycopene Extractions and Analysis by HPLC

β**-**carotene and lycopene were extracted from fruits and were analyzed by HPLC according to the method previously described by Xu et al. [73]. Extracts were analyzed on Agilent 1100 HPLC system equipped with a C18 Kromasil column (250 mm × 4.6 mm, 5 µm). The acetonitrile:methanol:dichloromethane ratio was 70:15:15 as the mobile phase, with a flow rate of 1.0 mL·min^−1^ isocratic elution and a 10 μL injection volume with a detection wavelength of 450 nm in 35 min. β**-**carotene and lycopene contents were calculated using a standard curve method and expressed as mg·kg^−1^ FW. There were three replicates for the measurement of β**-**carotene and lycopene.

### 4.3. RNA Extraction, cDNA Library Construction and Sequencing

Total RNA was extracted from each sample using the TRIzol reagent (Invitrogen, Carlsbad, USA) and DNase I was then used to remove DNA (Takara, Dalian, China). The quality, quantity, and integrity of the total RNA were evaluated using Nanodrop (IMPLEN, CA, USA), Qubit 2.0 (Life Technologies, CA, USA), and Aglient 2100 (Agilent Technologies, CA, USA). Briefly, the first cDNA chain was synthesized using random hexamers as a six-base random primer, and the second cDNA chain was synthesized by adding a buffer solution, dNTPs, RNase H, and DNA polymerase I. The purified double-stranded cDNAs were terminal repaired, tailed, and sequenced. Then, the fragment size was selected by AMPure XP (Beckman Coulter, Brea, CA, USA) beads and the cDNA library was obtained by PCR enrichment. Finally, libraries were loaded on Illumina/Solexa HiSeq2000 platform with a sequenced read length set to PE100.

### 4.4. Reads Assembly and Functional Annotation

The raw reads were filtered to remove adapter sequences and reads with low-quality sequences containing more than 10% of bases which had a Q-value (quality score) of ≤ 20. The clean reads were assembled de novo using the Trinity Method [74]. Using the longest transcription as a sample of unigene, nine samples of unigene were combined. To calculate unigene expression, we used the fragments per kilobase of transcript per million mapped reads (FPKM) method [75]. Unigenes differentially expressed between two samples were screened using a false discovery rate (FDR) of < 0.01 and |log_2_ (fold changes)| of ≥1 as the criteria. To get annotation unigene sequences, the BLASTX alignment (E-value < 10^−5^) was performed between unigenes and Nr, Swiss-Prot, KEGG, COG, GO databases. The Blast2GO program [76] was used to obtain GO annotations of the unigenes based on Nr annotations.

The RNA-Seq data were deposited in NCBI database (https://www.ncbi.nlm.nih.gov/sra/) [77] with an SRA accession number PRJNA517027.

### 4.5. Identification of Genes Related to the Carotenoid Biosynthesis Pathways and Transcription Factor

According to the selected candidate gene information, genes responsible for the biosynthetic pathway of carotenoid were searched using the annotation database. Furthermore, the Arabidopsis Information Resource (TAIR) and sweet orange (*C. sinensis* L. Osbeck) annotation databases were used to analyze the homologous sequence similarly of the structural genes. The identified and searched sequences were confirmed by the NCBI BLAST program [14]. In order to understand the underlying transcriptional mechanisms of three pummelo fruits, we used the transcription factor prediction module PlantTFDB 3.0 [78] with a default *E*-value of 10^−6^.

### 4.6. Correlation Analysis of Structural Genes and Transcription Factors

A correlation analysis of structural genes and transcription factors was carried out to identify transcription factors that were co-expressed with the functional genes involved in carotenoid biosynthesis. In order to exclude false positives, functional genes and transcription factors with an FPKM value of ≥10.0 in at least one of the three stages during fruit development were selected, and transcription factors with correlation coefficient values of ≥0.666 by the *t-*test (the formula to calculate *t* value was *t* = (*r*√(n–2))/√(1–r^2^), at *p* < 0.05 and *n* = 9. |*t*| > *t*_0.05,7_ = 2.365 means significant correlation, so *r* > 0.666 means significant correlation. The co-expression analysis was performed by “CORREL” function in “EXCEL 2003” and confirmed by an in-house Perl scripts and IBM SPSS Statistics software.

### 4.7. RT-qPCR analysis

To validate the reliability of our transcriptome dataset, 10 putative candidate functional genes and 12 transcription factors selected from the DEGs were further analyzed using RT-qPCR. Total RNA of nine fruit samples was extracted using the TRIzol^®^ Reagent (Invitrogen, USA). RT-qPCR was performed on a CFX96 real-time system (BIO-RAD, Hercules, CA, USA) with a TaKaRa 2×SYBR Premix ExTaq^TM^ kit (Takara, Dalian, China). RT-qPCR was performed under the following conditions: at 95 °C for 30 s, followed by 40 cycles at 95 °C for 5 s and at 72 °C for 30 s. Relative expression analysis was performed using the 2^−ΔΔCT^ method. The transcript levels were normalized relative to the actin gene. The RT-qPCR primer sequences are listed in Table S7. Three biological and three technical replicates were used for the RT-qPCR assays.

### 4.8. Experimental Design and Statistical Analysis

There were three biological replicates per cultivar for each sampling date and each biological replicate was mixed from at least five fruits. Considering that HRMY and HJMY were the bud mutation of GXMY and fruit samples at different development stages and used, one biological replicate of mixed sample was used for RNA-Seq in each time point for each cultivar. There were three replicates for the measurement of β**-**carotene, lycopene, and RT-qPCR assays. Differences among the nine samples were analyzed by three (cultivars) × three (sampling dates) ANOVA. Means were separated by the Duncan’s new multiple range test at *p* < 0.05.

## 5. Conclusions

Our results clearly showed that HJMY has the highest content of β-carotene, followed by HRMY and GXMY. Lycopene is dominantly accumulated in HRMY. By using RNA-Seq technique, we identified 357 genes involved in biosynthesis of secondary metabolites, of which 12 DEGs are involved in carotenoid biosynthesis. Among these 12 DEGs, *PSY2*, *LYCB2*, *LYCE*, *CCD4*, *NCED2*, *AAO3*, and *CYP707A1* are the most distinct DEGs in three pummelo cultivars. The co-expression analysis revealed that the expression patterns of several transcription factors such as *bHLH*, *MYB*, *ERF*, *NAC*, and *WRKY* are highly correlated with DEGs, which are involved in carotenoid biosynthesis. In addition, the expression patterns of 22 DEGs are validated by real-time RT-qPCR and the results are highly concordant with the RNA-Seq results. In conclusion, our results provide a global vision of transcriptomic profile among three pummelo cultivars with different pulp colors and would be beneficial to further study the molecular mechanism of carotenoid accumulation in pummelo flesh and help the breeding of citrus with high carotenoid content.

## Figures and Tables

**Table 1:** Summary of transcriptomic sequencing and de novo assembly.

Samples	Read number	Base number	GC content	% (≥Q30)
GXMY1 (40 DAF)	22,744,151	4,581,699,084	46.49%	85.92%
GXMY2 (100 DAF)	22,575,178	4,548,053,121	46.10%	85.79%
GXMY3 (190 DAF)	22,780,099	4,589,605,411	45.77%	85.82%
HRMY1 (40 DAF)	22,437,953	4,515,689,216	47.34%	85.73%
HRMY2 (100 DAF)	22,964,175	4,624,970,836	45.06%	86.05%
HRMY3 (190 DAF)	22,607,952	4,557,243,046	44.76%	85.88%
HJMY1 (40 DAF)	22,064,221	4,450,273,681	46.50%	85.68%
HJMY2 (100 DAF)	19,694,950	3,977,639,966	45.25%	85.02%
HJMY3 (190 DAF)	23,914,670	4,816,251,310	45.48%	86.02%
Total	201,783,349	40,661,425,671	45.86%	85.76%
After assembly				
Total clean reads	201,783,349			
Total clean nucleotides (nt)	40,661,425,671			
Total number of contigs	17,658,287			
Total length of contigs (nt)	656,824,421			
Mean length of contigs (nt)	37			
N50 of contigs	37			
Total number of unigenes	54,051			
Total length of unigenes (nt)	46,385,997			
Mean length of unigenes (nt)	858			
N50 of unigenes	1,585			

**Table 2:** Differentially expressed genes at fruit developmental stages in three pummelo cultivars.

Fruit development at different stages (days)	GXMY			HRMY			HJMY		
	ALL DEGs	Up-regulated	Down-regulated	ALL DEGs	Up-regulated	Down-regulated	ALL DEGs	Up-regulated	Down-regulated
40 DAF/100 DAF	3240	1068	2172	1887	371	1516	2080	421	1,659
40 DAF/190 DAF	3882	1408	2474	2569	753	1816	2316	639	1,677
100 DAF/190 DAF	2562	1300	1262	4988	1875	3113	3371	1796	1,575
40 DAF/100 DAF/190 DAF	616			258			256		

**Table 3:** Expression profiles of carotenoid biosynthesis-related genes at fruit developmental stages in three pummelo cultivars.

Gene Name	Unigene ID	Gene Length	GXMY			HRMY			HJMY		
			40 DAF	100 DAF	190 DAF	40 DAF	100 DAF	190 DAF	40 DAF	100 DAF	190 DAF
*PSY2*	c27977.graph_c0	1373	3.45	0.41	0.04	0.04	0.32	0	0	0.28	0.04
*LYCB2*	c35136.graph_c0	2037	5.57	15.15	19.49	2.3	15.07	25.57	0.58	15.4	19.32
*LYCE*	c29745.graph_c0	2018	6.22	4.01	1.64	0.71	6.95	4.68	0.63	7.78	3.09
*CCD1*	c29184.graph_c0	903	6.04	3.81	0.65	1.4	5.87	0.47	1.26	5.31	0
*CCD4*	c20564.graph_c0	3057	60.55	20.59	16.36	96.28	18.75	16.19	118.99	17.24	12.32
*NCED1*	c17328.graph_c1	1536	8.23	0.45	5.20	12.65	1.09	10.87	2.23	0.63	8.46
*NCED2*	c31914.graph_c0	2425	17.56	107.12	99.71	10.13	105.11	84.13	4.57	97.99	59.51
*NCED3*	c11942.graph_c0	1101	11.74	0.46	7.28	14.57	0.51	10.62	3.26	1.24	11.98
*AAO3*	c33740.graph_c0	4559	22.27	18.46	7.99	2.5	19.43	6.51	1.22	22.02	5.97
*CYP707A1*	c20275.graph_c0	2041	2.11	2.97	7.5	25.59	4.14	27.64	85.48	5.58	13.47
*CYP707A3*	c27956.graph_c0	1811	3.92	5.03	2.61	1.36	4.26	2.13	1.11	3.72	0.46
*CYP707A4*	c35953.graph_c0	1734	17.32	8.77	1.16	1.22	9.11	0.89	0.81	11.71	0.59

**Table 4:** Correlation analyses of structural genes involved in carotenoid metabolism and transcription factors.

Gene ID	FPKM (max)	FPKM (min)	Description for the best hit in *C. sinensis*	Number of correlations
c11225.graph_c0	17.95	0.69	bHLH107	4
12645.graph_c0	42.68	1.02	BIM1 isoform X1	3
c21405.graph_c0	3.45	0	bHLH162	2
c22086.graph_c0	9.37	0	bHLH57	2
c2272.graph_c0	2.60	0	bHLH35	2
c22910.graph_c0	14.15	0.46	bHLH61	4
c27501.graph_c0	21.44	5.78	bHLH130	4
c589.graph_c1	3.97	0	HEC2	2
c6149.graph_c0	2.74	0	ILR3	4
c34091.graph_c0	256.50	6.32	Ethylene-responsive transcription factor ERF12	3
c24274.graph_c0	12.57	1.31	Ethylene-responsive transcription factor-like protein	3
c34297.graph_c0	138.23	3.30	Ethylene-responsive transcription factor ERF107	3
c407.graph_c1	3.13	0	Ethylene-responsive transcription factor 13	2
c8122.graph_c0	111.06	5.19	Ethylene-responsive transcription factor RAP2-3	4
c8622.graph_c0	57.79	1.26	Ethylene-responsive transcription factor ERF23	2
c30160.graph_c0	22.31	0.04	AP2-like ethylene-responsive transcription factor ANT	1
c26695.graph_c0	2.83	0	AP2-like ethylene-responsive transcription factor AIL1	4
c30127.graph_c0	118.39	13.99	Auxin response factor 4	3
c27816.graph_c0	5.91	0	Growth-regulating factor 1	3
c9781.graph_c0	5.38	0	Growth-regulating factor 4	2
c22501.graph_c0	8.35	0.91	Transcriptional adapter ADA2b isoform X1	6
c23847.graph_c0	1296.81	106.33	MYB1R1-like	4
c25031.graph_c0	2.34	0	MYB11	4
c9240.graph_c0	6.37	0.20	MYB52	2
c1320.graph_c0	4.49	0	MYB13	2
c13169.graph_c0	104.34	1.68	Transcription factor AS1-like	6
c13009.graph_c0	157.18	2.18	REVEILLE 1	2
c21421.graph_c0	23.56	0.45	Transcription factor AS1	6
c13063.graph_c0	3047.44	242.76	Mini zinc finger protein 2	4
c21980.graph_c0	6.22	0.19	WRKY22	4
c34311.graph_c0	114.93	21.37	WRKY17	5
c13516.graph_c0	26.60	4.39	NAC72	3
c31393.graph_c0	20.63	3.05	Transcription factor RF2a-like	3
c28763.graph_c0	23.77	0.83	Transcription factor TGA7	3
c34869.graph_c0	58.15	5.27	BES1/BZR1 homolog protein 4	6
c35277.graph_c0	22.80	7.03	Transcription factor TCP20	3
c35957.graph_c0	17.19	1.35	Trihelix transcription factor GTL1	2
c31746.graph_c0	330.33	15.64	DELLA protein GAI	5
c31940.graph_c0	4.21	0	FAR1-related sequence 5-like	5
c28566.graph_c0	13.07	1.05	Transcription factor HHO2-like	6
c29740.graph_c0	26.66	1.14	B3 domain-containing transcription factor VRN1-like	4
c27534.graph_c0	303.99	13.66	Effector of transcription 2	4
c27216.graph_c0	110.23	3.11	Transcription factor TCP4	6
c22856.graph_c0	7.47	0	Trihelix transcription factor GT-2	4
c20987.graph_c0	30.86	0.03	GATA transcription factor 11	4
c14734.graph_c0	63.08	6.70	Transcription factor HHO3	5
c12838.graph_c0	49.59	9.91	YABBY 2 isoform X1	5
c10930.graph_c0	22.79	1.61	Trihelix transcription factor PTL	6

## Supplementary Materials

Supplementary materials can be found at https://www.mdpi.com/1422-0067/20/9/2246/s1. **Table S1.** List of KEGG pathway annotations in transcriptome of three pummelo cultivars. **Table S2.** The pathway enrichment analysis of DEGs during fruit development in three pummelo cultivars. **Table S3.** List of 435 transcription factor genes in three pummelo cultivars. **Table S4.** The number and percentage of transcription factors belonging to different families. **Table S5.** Differentially expressed transcription factor genes. **Table S6.** Correlation analysis of transcription factors and structural genes. **Table S7.** Primers sequences used for RT-qPCR analysis.
